# Evolutionary game and stability analysis of elderly care service quality supervision from the perspective of government governance

**DOI:** 10.3389/fpubh.2023.1218301

**Published:** 2023-08-22

**Authors:** Qiangxiang Wang, June Liu, Yue Zheng

**Affiliations:** Department of Logistics and E-Commerce, School of Economics and Management, Huaibei Normal University, Huaibei, China

**Keywords:** elderly care service, government governance, regulatory mechanism, evolutionary game, system dynamics

## Abstract

**Objective:**

The performance of government functions is an important guarantee for the standardized operation of the elderly service market. The objective of this study is to explore the optimal path for the government to govern the elderly care service market.

**Methods:**

The tripartite evolutionary game model is proposed in the paper, which composed of local governments, private elderly care institutions and the public. Furthermore, three mechanisms, i.e. dynamic penalty and static subsidy, static penalty and dynamic subsidy, dynamic penalty and dynamic subsidy, are designed. Under these different mechanisms, the stability of each subject’s strategy choice is analyzed by using system dynamics simulation.

**Results:**

The introduction of dynamic mechanisms can compensate for the inability of static mechanisms to bring the system to a steady state. The dynamic penalty and dynamic subsidy mechanism allows the system to evolve to the desired point of stability. The self-discipline behavior of private elderly care institutions is positively correlated with penalties and reputation gains-losses, negatively correlated with subsidies, and not correlated with supervision rewards. Excessive subsidies will promote the collusion of private elderly institutions.

**Conclusion:**

Only when the local government adopts the dynamic penalty and dynamic subsidy mechanism will private elderly care institutions choose to operate in a fully self-disciplined manner. Reasonable adjustments of penalties, reputation gains-losses and subsidies can not only further optimize the dynamic penalty and dynamic subsidy mechanism, but also help to achieve diversified regulatory objectives of the government. This study would provide a reference for local governments seeking to develop effective regulatory policies for the elderly service market.

## 1. Introduction

The entry of social capital into the elderly care industry can effectively solve the insufficient supply of elderly care services (1–4), improve the utilization efficiency of elderly care resources (5, 6), and meet the diversified, personalized, and hierarchical service needs of the older adult (7, 8). The role of social capital in the elderly service system cannot be ignored. In 2013, the Chinese government issued “Several Opinions on Accelerating the Development of the Elderly Service Industry,” proposing to improve the market mechanism and support social forces to run elderly institutions (9). In 2016, the Chinese government released “Several Opinions on Comprehensively Liberalizing the Elderly Care Service Market and Improving the Quality of Elderly Care Services,” which proposed to further relax the access conditions and optimize the market environment (10). In 2021, the Chinese government released the “14th Five-Year Plan for the Development of National Aging Cause and Elderly Care Service System,” which once again emphasized the need to fully mobilize the participation of social forces (11). However, as the role of social capital in the elderly service system gradually changes from “supplement” to “support,” the corresponding service quality problems also gradually appear. The late formation of China’s pension service market makes a series of top-level policy design relatively lagging behind, and the basic support for social capital is also insufficient. This makes it difficult to fundamentally improve the service quality of the elderly service market, and the problem of imperfect regulatory mechanisms still exists (12).

As is well-known, service quality is the necessary basis for the survival and development of elderly care institutions. The government supervision is an important guarantee for elderly care institutions to improve their service quality level. However, over the years, the high construction investment and low returns of private elderly care institutions have put the private elderly care institutions under greater operational pressure (13). This encourages them to pursue low-quality services that may reduce costs. For example, in 2015, a private elderly care institution in Henan Province cut corners in the construction of firefighting infrastructure to save costs, leading to a major fire (14). In 2020, another private elderly care institution in Sichuan Province did not provide services for the older adult according to the contracted content (15). In 2022, some private elderly care institutions in Shandong Province failed the sampling inspection of food service (16). The speculative operation of private elderly care institutions not only infringed on the legitimate rights and interests of the older adult, but also increased the difficulty of government supervision. On the one hand, due to the particularity of service, the speculative operation of private elderly care institutions is not easy to be observed directly. On the other hand, the existing government supervision mechanism is also difficult to fundamentally eliminate the speculative operation of private elderly care institutions. Therefore, it is urgent for the government to establish an effective regulatory mechanism to promote the healthy and sustainable development of the elderly service industry.

Previous studies are mainly focused on the supervision of the elderly care service market and the coping strategies of various elderly care service problems. Based on the background of “Internet and community elderly care,” Wang et al. (17) constructed a four-party evolutionary game model. The research results showed that the collaborative supervision of government and seniors has a greater impact on the behavior strategy of suppliers and platforms. Krings et al. (18) analyzed China’s aging dilemma and aging policies, noted that the Chinese government’s aging policies are similar to those of Western countries and are converge toward home care, informal care, and healthy aging. Hung (19) assessed the gaps in smart home care technology and policy formulation that Chinese policymakers need to address. Lopreite et al. (20, 21) studied the relationship between aging and health expenditure, and proposed that government policies should balance economic, social, and health in order to improve the quality of life of older adults in their later years and achieve sustainable economic development. Pförtner et al. (22) and Hower et al. (23) pointed out that nursing facilities are facing challenges such as rising costs and staff shortages during the COVID-19 epidemic, and timely actions should be taken to ensure the provision of long-term care services. Song et al. (24) studied the market-based transformation of elderly care services and founded that active government response to public needs and preferences and the establishment of market regulation measures can be effective in improving social welfare. Lim (25) concluded that government supervision and education are necessary to strengthen the professionalism of nursing staff through in-depth interviews with 10 nursing staff. The above studies are from the perspective of government, and less consideration is given to other stakeholders in the supply of elderly care services. Furthermore, most of the studies on regulation focus on the supervision objectives with the best results, but lack of consideration of supervision objectives such as improving market robustness and speeding up market stability.

The static reward and static punishment mechanism are used to discuss the impact of government penalties and rewards on the market. For example, Li et al. (26) developed a game model of the evolution of the governance mechanism of the recycling industry and analyzed the effect of government penalty intensity on the behavioral strategies of recycling firms. Zhu et al. (27) introduced the random punishment mechanism into the treatment of kitchen waste. The results verified that the random punishment mechanism is more conducive to the system to reach a stable state. Wang et al. (28) designed a penalty linkage mechanism and founded that the penalty linkage mechanism can promote the development of the electronic recycling industry in the initial stage. In addition, the dynamic reward and dynamic punishment mechanism are also established. For example, Sun et al. (29) analyzed the impact of pure reward and reward-dominated penalty-supplemented schemes on the behavior choices of game subjects. Based on the background of carbon emission reduction. He et al. (30) proposed an evolutionary game model to study the government’s regulatory strategy on straw biomass energy supply chain, in which that the dynamic penalty strategy is conducive to the sustainable development of biomass energy supply chain. The reward and punishment, as the intervention means often used by the government to regulate the market, have good incentive and restraint effects, but the proposed dynamic reward and dynamic punishment mechanism are mainly based on the government and the private sector. Also, they are lack of discussion on the dynamic reward and dynamic punishment mechanism under the participation of multiple agents, and consideration on the non-linear dynamic reward or punishment mechanism. This is not conducive to the depiction of the reality of the market.

As the end consumers of various products and services, the supervision behavior of the public is undoubtedly an important factor affecting the quality of products and services, and also another important topic related to this study. Through analysis, Zhou et al. (31) found that the establishment of a supervision mechanism with public participation can control the non-green behavior of enterprises and increase their demand for renewable energy. Feng et al. (32) introduced the public monitoring into the railroad transportation safety regulation model, and the results showed that the increase in the proportion of public monitoring was beneficial to improve the proportion of railroad safety production. Liu et al. (33) argued that the public’s negative participation in monitoring is not conducive to government monitoring of the green building supply market. Dong et al. (34) explored the impact of public supervision on industrial pollution control from the perspective of the government. Yang et al. (35) constructed an evolutionary game model consisting of regulators, energy companies and whistleblowers in which each party’s strategy choice is analyzed. Note that, the existing literature on the supervision of pension services are mainly focused on the regulatory role of the government and the older adult, which ignores that the public is an indispensable participant.

As is well-known, evolutionary game theory (EGT) (36) is often used to quantify the government’s intervention measures. Some scholars have discussed the relationship among the actors in the senior care service market by building evolutionary game model. For example, Sun et al. (37) developed an evolutionary game model to analyze the effect of government subsidies on the cooperation strategies of health care organizations and elderly social care organizations. Zhang and Yao (38) took the government as one of the game subjects to explore the inhibitory effect of government regulation on the speculative behavior of health-care PPP project operators. Yue and Lin (39) constructed a two-party game model to analyze the impact of punishment and operation subsidy on the quality supervision of pension PPP projects. Wang and Cui (40) used the EGT to analyze the different reward and punishment mechanisms between local governments and elderly care institutions, and pointed out that the dynamic reward mechanism is more conducive to the elderly care institutions to adopt self-discipline management behavior. Therefore, EGT is also suitable for the study of elderly care service quality supervision under multi-subject supervision.

In summary, the existing literature mainly discusses the government participation in the supervision of the pension service market and the game relationship among the main bodies in the pension service market. There is a lack of in-depth discussion on the differences of different combinations of subsidies and penalties under the public participate in supervision. On the other hand, the literature analyzing the government’s intervention behavior on the service supply behavior of private elderly care institutions mostly focuses on static reward and static penalty mechanism, with little consideration of non-linear dynamic reward and dynamic penalty mechanism. What is more, there is no consideration of the study of the relevant parameters adjustment priorities from the different regulatory objectives of the government.

Therefore, the contributions of this paper are summarized as follows. Firstly, considering the supervision behavior of the public, the dynamic reward and penalty mechanism is proposed, which is more appropriate to the actual situation. Secondly, from the different regulatory objectives of the government, the priorities of the relevant parameters of adjustment are studied to facilitate the government to prescribe the right medicine. Thirdly, a non-linear dynamic subsidy method is proposed, in which there is a parabolic relationship between subsidy and self-discipline management willingness of private elderly care institutions. This can more truly reflect the government’s subsidy behavior. Note that, the nonlinear dynamic subsidy method is similar to literature (41). However, there are differences in the following three aspects. (i) Our paper presents the static penalty and dynamic subsidy mechanism which is compared with other mechanisms (for details, see Section 4). (ii) In this paper, the public monitoring behavior is introduced. Furthermore, the impacts of different behavior strategies of private elderly care institutions on the older adult are quantified. (iii) This paper examines the priority of adjustment of relevant parameters from different regulatory objectives of the government. It may provide a decision-making reference for the government’s policy regulation.

## 2. Model assumptions and construction

### 2.1. Model assumptions

#### 2.1.1. Hypothesis 1: subject assumption

The local governments, the private elderly care institutions and the public are the subjects of the game. Assume that they are bounded rationality. Both the private elderly care institutions and the public pursue the maximization of their own interests, while local governments pursue the maximization of comprehensive social benefits.

#### 2.1.2. Hypothesis 2: strategy assumption

In the supply process of elderly care services, the probability of private elderly care institutions supplying high-quality service (HQ) or low-quality service (LQ) is (e, 1−e). The probability of local governments enforcing positive supervision (PS) or negative supervision (NS) is (g, 1−g) . The probability of the public choosing to participate in supervision (IS) or not to participate in supervision (OS) is (p,1−p) . Here, e,g,p∈[0, 1] .

#### 2.1.3. Hypothesis 3: parameter assumption for private elderly care institutions

The profit of private elderly care institutions supplying HQ is *R_h_*, and the profit of supplying LQ is *R_l_*. When the private elderly care institutions supply HQ or LQ, they will face reputation gains *R_e_* or losses *L_e_* respectively. Moreover, the action of supplying HQ will bring welfare *W_g_* to the society, and on the contrary, it will cause losses *D_g_* to the society.

#### 2.1.4. Hypothesis 4: parameter assumption for local governments

The cost of PS by local governments is *C_g_*. When the local governments enforce PS or NS, they will face credibility gains *R_g_* or losses *L_g_*, respectively. Moreover, the local governments will subsidize private elderly care institutions that supply HQ and punish private elderly care institutions that supply LQ, with values of S and *F_e_*, respectively.

#### 2.1.5. Hypothesis 5: parameter assumption for public

The cost of public choose IS is *C_p_*, and there is no cost if they choose OS. The public choose IS will be rewarded *R_p_* by the local governments. The change of social welfare and social loss will have an impact on the older adult who need elderly care services in the public. Therefore, the public will feel social welfare or social loss with a probability of α . The parameter symbols and their meanings are shown in Table 1.

**Table 1:** Parameter symbols and meanings.

Meaning	Parameter	Meaning	Parameter
Probability of private elderly care institutions supply high-quality service	*e*	The cost of local governments enforce positive supervision	*C_g_*
Probability of local governments enforce positive supervision	*g*	The credibility gains of local governments’ implementation of positive supervision	*R_g_*
Probability of public choose to participate in supervision	*p*	The credibility losses of local governments’ implementation of negative supervision	*L_g_*
The profit of private elderly care institutions supply high-quality service	*R_h_*	Government subsidies to private elderly care institutions that provide high-quality service	*S*
The profit of private elderly care institutions supply low-quality service	*R_l_*	Government penalties to private elderly care institutions that provide low-quality service	*F_e_*
Reputation gains of private elderly care institutions supply high-quality service	*R_e_*	The cost of public choose to participate in supervision	*C_p_*
Reputation losses of private elderly care institutions supply low-quality service	*L_e_*	Government rewards to the public who participate in supervision	*R_p_*
Social welfare brought by private elderly care institutions supplying high-quality service	*W_g_*	Probability of public feel social welfare or social loss	α
Social loss brought by private elderly care institutions supplying low-quality service	*D_g_*		

### 2.2. Model construction

According to the aforementioned hypotheses, the payment matrix of private elderly care institutions, local governments and the public is constructed, which is shown in Table 2.

**Table 2:** Payment matrix for all players.

		Local governments	Public	
			IS (*p*)	OS (1 − *p*)
**Private elderly care institutions**	**HQ** (e)	**PS** (g)	$R_{h}+R_{e}+S$	$R_{h}+R_{e}+S$
			$W_{g}+R_{g}-C_{g}-S$	$W_{g}+R_{g}-C_{g}-S$
			$\alpha W_{g}-C_{p}$	$\alpha W_{g}$
		**NS** (1−g)	$R_{h}+R_{e}$	$R_{h}+R_{e}$
			$W_{g}-L_{g}$	$W_{g}-L_{g}$
			$\alpha W_{g}-C_{p}$	$\alpha W_{g}$
	**LQ** (1−e)	**PS** (g)	$R_{l}-L_{e}-F_{e}$	$R_{l}-L_{e}-F_{e}$
			$F_{e}+R_{g}-D_{g}-C_{g}-R_{p}$	$F_{e}+R_{g}-D_{g}-C_{g}$
			$R_{p}-\alpha D_{g}-C_{p}$	$-\alpha D_{g}$
		**NS** (1−g)	$R_{l}-L_{e}-F_{e}$	$R_{l}-L_{e}$
			$F_{e}-D_{g}-L_{g}-R_{p}$	$-D_{g}-L_{g}$
			$R_{p}-\alpha D_{g}-C_{p}$	$-\alpha D_{g}$

It follows from payment matrix that, when private elderly care institutions choose to supply high-quality services (low-quality services), their expected revenue is *U_e_* (*U*_1−*e*_):

(1) $$U_{e}=R_{h}+R_{e}+gS$$

(2) $$U_{1-e}=R_{l}-L_{e}-gF_{e}-\left(1-g\right)pF_{e}$$

The average expected revenue of the private elderly care institutions is proposed as follows:

(3) $${\bar{U}}_{e}=eU_{e}+\left(1-e\right)U_{1-e}$$

From (1)–(3), it can be seen that the replicator dynamic equation of the private elderly care institutions can be formulated as follows:

(4) $$\begin{array}{l} F\left(e\right)=de/dt=e\left(U_{e}-{\bar{U}}_{e}\right)=\\ \;\;\;\;\;\;e\left(1-e\right)\left[R_{h}+R_{e}+gS-R_{l}+L_{e}+gF_{e}+\left(1-g\right)pF_{e}\right] \end{array}$$

Similarly, the replicator dynamic equation of the local governments can be written:

(5) $$\begin{array}{l} F\left(g\right)=dg/dt=\\ \;\;\;\;\;g\left(1-g\right)\left[R_{g}-C_{g}-eS+L_{g}+\left(1-e\right)\left(1-p\right)F_{e}\right] \end{array}$$

The replicator dynamic equation of the public can be formulated as follows:

(6) $$F\left(p\right)=dp/dt=p\left(1-p\right)\left[\left(1-e\right)R_{p}-C_{p}\right]$$

It follows from the equations (4)–(6) that, a three-dimensional replicator dynamic system (I) can be obtained as follows:

(7) $$\left\{ \begin{array}{l} F\left(e\right)=e\left(1-e\right)\left[R_{h}+R_{e}+gS-R_{l}+L_{e}+gF_{e}+\left(1-g\right)pF_{e}\right]\\ F\left(g\right)=g\left(1-g\right)\left[R_{g}-C_{g}-eS+L_{g}+\left(1-e\right)\left(1-p\right)F_{e}\right]\\ F\left(p\right)=p\left(1-p\right)\left[\left(1-e\right)R_{p}-C_{p}\right] \end{array}\right.$$

## 3. Model analysis

### 3.1. Model stability

Let all equations in the replicator dynamic system (I) be equal to 0. The following nine potential equilibrium points *E*_1_(0, 0, 0), *E*_2_(0, 0, 1), *E*_3_(0, 1, 0), *E*_4_(1, 0, 0), *E*_5_(0, 1, 1), *E*_6_(1, 0, 1), *E*_7_(1, 1, 0), *E*_8_(1, 1, 1), and *E*_9_(e*, g*, p*) can be obtained. Note that the equilibrium point of a game system is not always stable. Therefore, this paper will use the Lyapunov’s first method and the Jacobian matrix to analyze the stability of each equilibrium point. The corresponding Jacobian matrix is stated as follows:

(8) $$J=\left[\begin{array}{ccc} \partial F\left(e\right)/\partial e & \partial F\left(e\right)/\partial g & \partial F\left(e\right)/\partial p\\ \partial F\left(g\right)/\partial e & \partial F\left(g\right)/\partial g & \partial F\left(g\right)/\partial p\\ \partial F\left(p\right)/\partial e & \partial F\left(p\right)/\partial g & \partial F\left(p\right)/\partial p \end{array}\right]$$

According to Lyapunov’s first method, if all eigenvalues of the Jacobian matrix have negative real parts, the equilibrium point is an evolutionary stable strategy (ESS); if the eigenvalues of the Jacobian matrix have at least one positive real part, then the equilibrium point is an instability point; if some real parts of the eigenvalues of the Jacobi matrix are 0 and the rest are negative, then the equilibrium point is in a critical state and stability cannot be judged. Stability analysis of all equilibrium points is shown in Table 3. It can be seen from Table 3 that, the equilibrium points *E*_6_(1, 0, 1) and *E*_8_(1, 1, 1) are not the stable strategies of the evolutionary game, and the stability of the remaining seven equilibrium points cannot be directly judged. Therefore, further research will be carried out via system dynamics method.

**Table 3:** Stability analysis of all equilibrium points.

Equilibrium point	Eigenvalue λ_1_, λ_2_, λ_3_	Symbol judgment	Results
*E*_1_(0, 0, 0)	$R_{h}+R_{e}-R_{l}+L_{e}$ , $R_{g}-C_{g}+L_{g}+F_{e}$ , $R_{p}-C_{p}$	×××	Uncertainty
*E*_2_(0, 0, 1)	$R_{h}+R_{e}-R_{l}+L_{e}+F_{e}$ , $R_{g}-C_{g}+L_{g}$ , $C_{p}-R_{p}$	×××	Uncertainty
*E*_3_(0, 1, 0)	$R_{h}+R_{e}+S-R_{l}+L_{e}+F_{e}$ , $C_{g}-R_{g}-L_{g}-F_{e}$ , $R_{p}-C_{p}$	×××	Uncertainty
*E*_4_(1, 0, 0)	$R_{l}-R_{h}-R_{e}-L_{e}$ , $R_{g}-C_{g}-S+L_{g}$ , $-C_{p}$	××−	Uncertainty
*E*_5_(0, 1, 1)	$R_{h}+R_{e}+S-R_{l}+L_{e}+F_{e}$ , $C_{g}-R_{g}-L_{g}$ , $C_{p}-R_{p}$	×××	Uncertainty
*E*_6_(1, 0, 1)	$R_{l}-R_{h}-R_{e}-L_{e}-F_{e}$ , $R_{g}-C_{g}-S+L_{g}$ , $C_{p}$	××+	Instability
*E*_7_(1, 1, 0)	$R_{l}-R_{h}-R_{e}-S-L_{e}-F_{e}$ , $C_{g}+S-R_{g}-L_{g}$ , $-C_{p}$	××−	Uncertainty
*E*_8_(1, 1, 1)	$R_{l}-R_{h}-R_{e}-S-L_{e}-F_{e}$ , $C_{g}+S-R_{g}-L_{g}$ , $C_{p}$	××+	Instability
*E*_9_(e*, g*, p*)	λ_1_*, λ_2_*, λ_3_*	×××	Uncertainty

× , Uncertainty of positive or negative; + , Eigenvalues are positive; and − , Eigenvalues are negative.

### 3.2. System dynamics model construction

**Figure 1:**
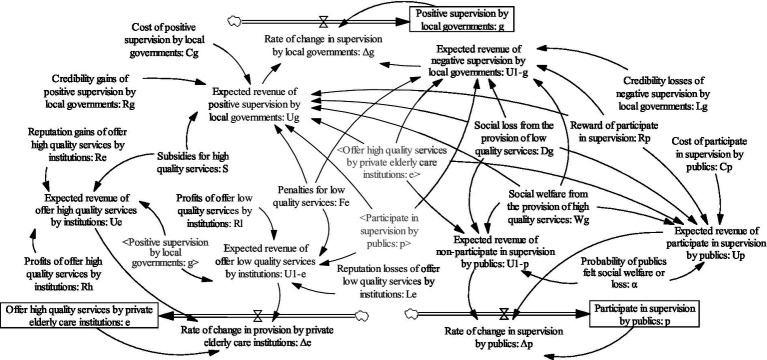
System dynamics model of elderly service market supervision.

System dynamics (42) is a scientific method which is often used to analyze the dynamic relationships among system function, structure, feedback, and behavioral strategies. It also can effectively depict the strategic choice of stakeholders from the perspective of system. Recently, some scholars have combined the evolutionary game theory with the system dynamics theory, and achieved good results (43–48) Referring to the model assumptions and replicated dynamic equations in the previous section, a system dynamics model is constructed which consists of three parties, i.e., local governments, private elderly care institutions, and the public, as shown in Figure 1.

The initial conditions of the system dynamics model are set as: INITIALTIME=0, FINALTIME=100, TIMESTEP=0.05 (41). In 2021, the number of elderly care institutions in Beijing increased to 579 in which 458 elderly care institutions have obtained star qualifications. Thus, the initial value of e is set to 0.79. The values of g and p are, respectively, set to 0.20 and 0.10 with reference to literature (49). According to the “Measures for the Management of Operating Subsidies for Elderly Care Institutions in Beijing,” elderly care institutions that meet the star rating standards can receive subsidies ranging from 600, 1,200, and 1,800 yuan per bed per year. In particular, two-star elderly care institutions, which account for nearly 70% of all star-qualified pension institutions, can receive a subsidy of 600 yuan per bed per year. Therefore, taking two-star elderly care institutions as an example, based on a single elderly care institution with 300 beds and a bed utilization rate of 46.71% (50). The total annual subsidy received by all elderly care institutions in Beijing is RMB 38.51 million, i.e., S=0.385 . The average annual profit of a single elderly care institutions in Beijing is RMB 0.9151 million, so the total annual profit of all elderly care institutions in Beijing is RMB 52984 million. Then, *R_h_* is about 5.298. In general, the speculative profits is higher than self-regulated profits. With reference to the literature (41), set *R_l_* = 6.248. According to the “Report on the Development of Aging in Beijing (2021),” the registered population aged 60 and above in Beijing is 3.883 million, accounting for 27.5% of the total registered population. Taking the proportion of the older adult in the total population as the probability of social welfare or loss felt by the public, it is easily obtained that α=0.275 . The specific parameters and initial value are shown in Table 4.

**Table 4:** Parameter assignment.

Parameter	Initial value	Parameter	Initial value
*e*	0.79	*C_g_*	0.6
*g*	0.20	*R_g_*	0.3
*p*	0.10	*L_g_*	0.3
*R_h_*	5.298	*S*	0.385
*R_l_*	6.248	*F_e_*	0.61
*R_e_*	0.1	*C_p_*	0.2
*L_e_*	0.2	*R_p_*	0.18
*W_g_*	0.3	α	0.275
*D_g_*	0.4		

**Figure 2:**
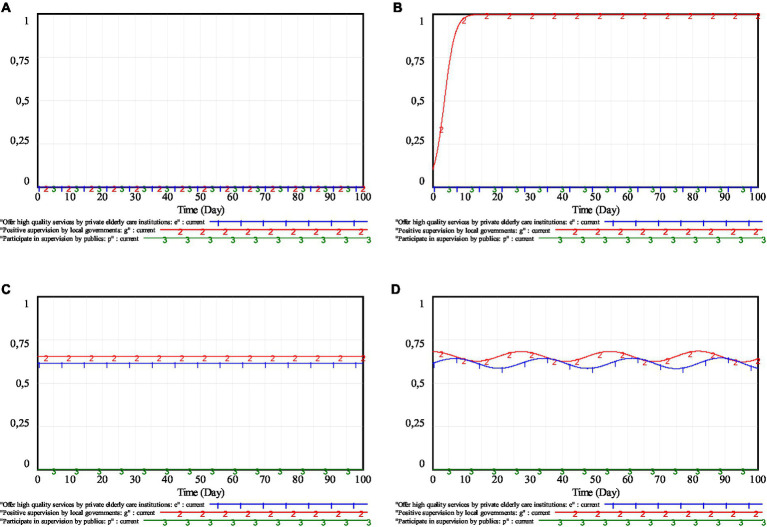
Evolutionary trajectories of equilibrium points for pure and mixed strategies. **(A)** Simulation result of pure strategy equilibrium point *E*_1_; **(B)** Simulation result of *E*_1_ mutation (*g* = 0 → 0.1); **(C)** Simulation result of mixed strategy equilibrium point *E*_10_; **(D)** Simulation result of *E*_10_ mutation (*g* = 0.6533 → 0.68).

Under the initial parameters, Lyapunov’s first method is used again to judge the stability of each equilibrium point, and the results are shown in Table 5. From Table 5, it can be seen that, under the static penalty and static subsidy mechanism, all equilibrium points of the system are not ESS. Taking the pure strategy equilibrium point *E*_1_(0, 0, 0) and the mixed strategy equilibrium point *E*_10_(0.6131, 0.6533, 0) as examples, the simulation results are shown in Figure 2. As can be seen from Figures 2A,C, the tripartite game players have reached a relatively balanced state under the corresponding initial strategies. However, this does not imply that the state is stable. As long as one or more players in the system fine-tune their strategies, the system will lose its original equilibrium state. For example, if the strategy proportion of the local governments in Figures 2B,D is adjusted to 0.1 and 0.68, respectively, the equilibrium state of the original system will be broken. Similarly, equilibrium points *E*_2_, *E*_3_, *E*_4_, *E*_5_, *E*_6_, *E*_7_, *E*_8_, and *E*_9_, are also unstable.

**Table 5:** Stability analysis of all equilibrium points.

Equilibrium point	Eigenvalue λ_1_, λ_2_, λ_3_	Results
*E*_1_(0, 0, 0)	(−0.65, 0.61, −0.02)	Saddle point
*E*_2_(0, 0, 1)	(−0.04, 0, 0.02)	Saddle point
*E*_3_(0, 1, 0)	(0.345, −0.61, −0.02)	Saddle point
*E*_4_(1, 0, 0)	(0.65, −0.385, −0.2)	Saddle point
*E*_5_(0, 1, 1)	(0.345, 0, 0.02)	Saddle point
*E*_6_(1, 0, 1)	(0.04, −0.385, 0.2)	Saddle point
*E*_7_(1, 1, 0)	(−0.345, 0.385, −0.2)	Saddle point
*E*_8_(1, 1, 1)	(−0.345, 0.385, 0.2)	Saddle point
*E*_9_(0, 0.1039, 1)	(0.000002, 0, 0.02)	Saddle point
*E*_10_(0.6131, 0.6533, 0)	(0.000001 + 0.0769i, 0.000001–0.0769i, −0.130358)	Saddle point

## 4. Regulatory mechanism optimization and stability analysis

Inspired by literature (41), three regulatory mechanisms, i.e., dynamic penalty and static subsidy, static penalty and dynamic subsidy, and dynamic penalty and dynamic subsidy, are proposed. Furthermore, both the advantages and disadvantages of them are discussed in the context of specific scenarios.

### 4.1. Stability analysis of dynamic penalty and static subsidy mechanism

**Figure 3:**
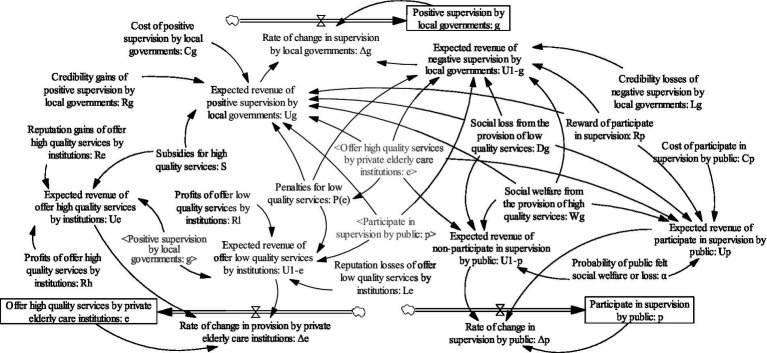
System dynamics model under dynamic penalty and static subsidy mechanism.

In the supervision of the elderly service market, the service supply behavior of private elderly care institutions often depends on the corresponding consequences that speculative operation may face. Therefore, the amount of government penalties should be adjusted according to the willingness of private elderly care institutions to operate in a self-disciplined manner. Assuming that the dynamic penalty function is $P\left(e\right)=F_{e}\left(1-e\right)+\varepsilon ,$ where ε is a constant. The optimal value of ε can be obtained by simulation debugging as ε=0.82 . The system dynamics model under the dynamic penalty and static subsidy mechanism is shown in Figure 3.

**Figure 4:**
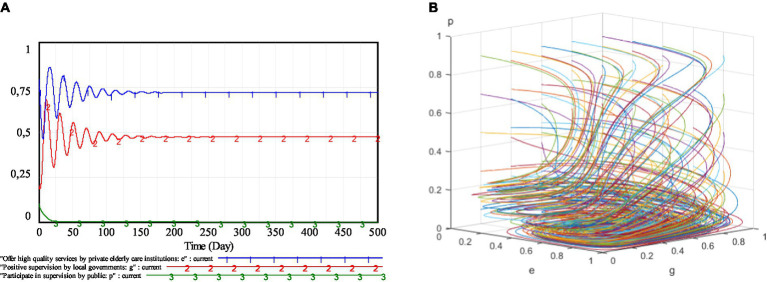
Simulation results under dynamic penalty and static subsidy mechanism. **(A)** (*e, g, p*) = (0.79, 0.2, 0.1); **(B)** Different initial willingness.

Under the initial simulation parameters, the initial willingness of the three parties is (0.79, 0.2, 0.1), and FINALTIME=500 is set at the same time to observe the convergence results of the system more clearly. The simulation result is shown in Figure 4A, which shows that the system is stable at point (0.7201, 0.4725, 0). Changing the initial willingness of the three parties, with other conditions keep constant, the ESS of the system can also be obtained as (0.7201, 0.4725, 0; Figure 4B). It implies that, under the dynamic penalty and static subsidy mechanism, the strategy selection of the tripartite game subjects will gradually stabilize, and eventually stabilize at point (0.7201, 0.4725, 0).

#### 4.1.1. Proposition 1

The dynamic penalty and static subsidy mechanism can bring the strategy choice of each subject in the regulation of the elderly service market to a steady state and stabilize at point (0.7201, 0.4725, 0).

##### 4.1.1.1. Proof

Substituting P(e) into the three-dimensional replicator dynamic system (I), we can get a new three-dimensional replicator dynamic system (II):

(9) $$\left\{ \begin{array}{l} F\left(e\right)=e\left(1-e\right)\left[R_{h}+R_{e}+gS-R_{l}+L_{e}+gP\left(e\right)+\left(1-g\right)pP\left(e\right)\right]\\ F\left(g\right)=g\left(1-g\right)\left[R_{g}-C_{g}-eS+L_{g}+\left(1-e\right)\left(1-p\right)P\left(e\right)\right]\\ F\left(p\right)=p\left(1-p\right)\left[\left(1-e\right)R_{p}-C_{p}\right] \end{array}\right.$$

Let all equations in the three-dimensional replicator dynamic system (II) be equal to 0. Then, nine new equilibrium points, i.e., *E*_1_*_a_*(0, 0, 0), *E*_2_*_a_*(0, 0, 1), *E*_3_*_a_*(0, 1, 0), *E*_4_*_a_*(1, 0, 0), *E*_5_*_a_*(0, 1, 1), *E*_6_*_a_*(1, 0, 1), *E*_7_*_a_*(1, 1, 0), *E*_8_*_a_*(1, 1, 1), and *E*_9_*_a_*(0.7201, 0.4725, 0) can be obtained. The equilibrium points *E*_1_*_a_* ~ *E*_8_*_a_* are substituted into the Jacobian matrix $J^{\prime }$ in turn, and the eigenvalues obtained contain the situation greater than 0. So the equilibrium points *E*_1_*_a_* ~ *E*_8_*_a_* are not ESS. Substituting the mixed equilibrium point *E*_9_*_a_* into the Jacobian matrix, it can be obtained that:

(10) $$J^{\prime }=\left[\begin{array}{ccc} -0.058110 & 0.277288 & 0.105336\\ -0.385450 & 0.000004 & -0.053456\\ 0 & 0 & -0.149618 \end{array}\right]$$

It is easily calculate that the eigenvalues of the mixed equilibrium point *E*_9_*_a_* are $\lambda_{1}=-0.0291+0.3256i,$ $\lambda_{2}=-0.0291-0.3256i,$ and $\lambda_{3}=-0.1496$ respectively. These eigenvalues are all less than 0, so the ESS of the system under the dynamic penalty and static subsidy mechanism is *E*_9_*_a_*(0.7201, 0.4725, 0).

Proposition 1 suggests that, the dynamic penalty and static subsidy mechanism adopted by the local governments can restrain the fluctuation of the strategy choice of the relevant game subjects and make the system reach a stable state. However, there are still some shortcomings in this mechanism, and private elderly care institutions still have a certain probability of choosing to provide low-quality services.

### 4.2. Stability analysis of static penalty and dynamic subsidy mechanism

**Figure 5:**
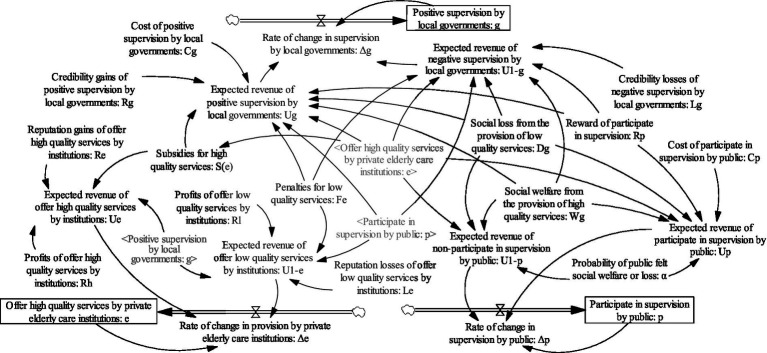
System dynamics model under static penalty and dynamic subsidy mechanism.

In general, penalty and subsidy are two main ways for local governments to restrain the elderly service market. China’s policy stipulates that all elderly care institutions that meet the quality standards can enjoy subsidies. Then the dynamic subsidy function can also be designed according to the supply willingness of private elderly care institutions. However, unlike dynamic penalty, local governments are constrained by costs and tend to relax incentives after achieving the desired goals. Therefore, the dynamic subsidy function can be designed as $S\left(e\right)=-e^{2}+\theta e+\omega$ , where θ and ω are constants. The system dynamics model under the static penalty and dynamic subsidy mechanism is shown in Figure 5.

**Figure 6:**
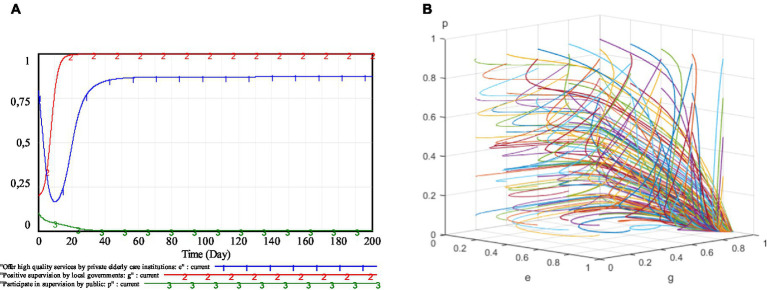
Simulation results under static penalty and dynamic subsidy mechanism. **(A)** (*e, g, p*) = (0.79, 0.2, 0.1); **(B)** Different initial willingness.

Assume that the initial willingness of the three game subjects is (0.79, 0.2, 0.1), and set θ=0.8 , ω=0.1 , FINALTIME=200. The simulation results of specific willingness and different initial willingness are shown in Figure 6. From Figure 6, it can be seen that, under the static penalty and dynamic subsidy mechanism, the strategy evolution trajectory of each game subject will change as the initial willingness of the relevant game subjects is adjusted, but the system will eventually stabilize at (0.869, 1, 0).

#### 4.2.1. Proposition 2

The static penalty and dynamic subsidy mechanism can bring the strategy choice of each subject in the regulation of the elderly service market to a steady state and stabilize at point (0.869, 1, 0).

##### 4.2.1.1. Proof

Replacing S with S(e) and substituting it into the three-dimensional replicator dynamic system (I), we can obtain the three-dimensional replicator dynamic system (III) as follows:

(11) $$\left\{ \begin{array}{l} F\left(e\right)=e\left(1-e\right)\left[R_{h}+R_{e}+gS\left(e\right)-R_{l}+L_{e}+gF_{e}+\left(1-g\right)pF_{e}\right]\\ F\left(g\right)=g\left(1-g\right)\left[R_{g}-C_{g}-eS\left(e\right)+L_{g}+\left(1-e\right)\left(1-p\right)F_{e}\right]\\ F\left(p\right)=p\left(1-p\right)\left[\left(1-e\right)R_{p}-C_{p}\right] \end{array}\right.$$

It follows from the three-dimensional replicator dynamic system (III) that, nine new equilibrium points can be obtained. These new equilibrium points are *E*_1_*_b_*(0, 0, 0), *E*_2_*_b_*(0, 0, 1), *E*_3_*_b_*(0, 1, 0), *E*_4_*_b_*(1, 0, 0), *E*_5_*_b_*(0, 1, 1), *E*_6_*_b_*(1, 0, 1), *E*_7_*_b_*(1, 1, 0), *E*_8_*_b_*(1, 1, 1), and *E*_9_*_b_*(0.869, 1, 0). Substituting the equilibrium point *E*_9_*_b_* into the Jacobian matrix $J^{\prime \prime }$ , the equation (12) can be *E*_9_*_b_* obtained. According to equation (12), the eigenvalues corresponding to equilibrium point can be calculated as $\lambda_{1}=-0.1068$ , $\lambda_{2}=-0.0451$ , $\lambda_{3}=-0.1764$ respectively. Because of $\lambda_{1},\lambda_{2},\lambda_{3}<0$ , so equilibrium point *E*_9_*_b_* is ESS. Similarly, equilibrium points *E*_1_*_b_* ~ *E*_8_*_b_* are not ESS.

(12) $$J^{\prime \prime }=\left[\begin{array}{ccc} -0.10681 & 0.074 & 0\\ 0 & -0.045116 & 0\\ 0 & 0 & -0.17642 \end{array}\right]$$

Proposition 2 suggests that, local governments can also make the system reach a stable state by adopting static penalty and dynamic subsidy mechanism. However, under this mechanism, private elderly care institutions still have a certain probability of choosing to provide low-quality services. Therefore, further optimization of the government regulatory mechanism is needed.

### 4.3. Stability analysis of dynamic penalty and dynamic subsidy mechanism

**Figure 7:**
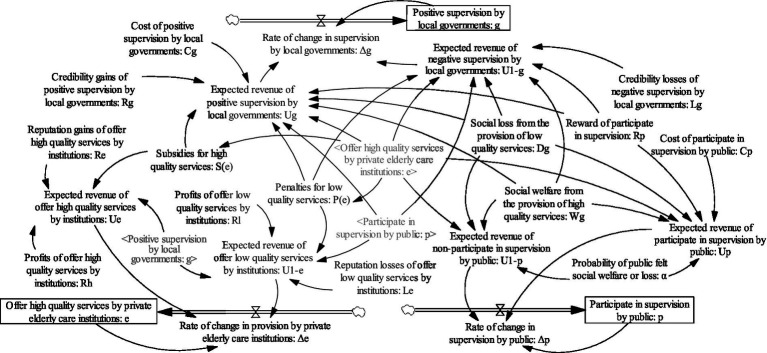
System dynamics model under dynamic penalty and dynamic subsidy mechanism.

Clearly, both the dynamic penalty and static subsidy mechanism and the static penalty and dynamic subsidy mechanism are conducive to local government’s supervision of the elderly service market. However, these two mechanisms cannot completely eliminate the speculative operation of private elderly care institutions. Therefore, a dynamic penalty and dynamic subsidy mechanism is further designed. It consists of the dynamic penalty function $P\left(e\right)=F_{e}\left(1-e\right)+0.82$ and the dynamic subsidy function $S\left(e\right)=-e^{2}+0.8e+0.1.$ The system dynamics model under the dynamic penalty and dynamic subsidy mechanism is shown in Figure 7.

**Figure 8:**
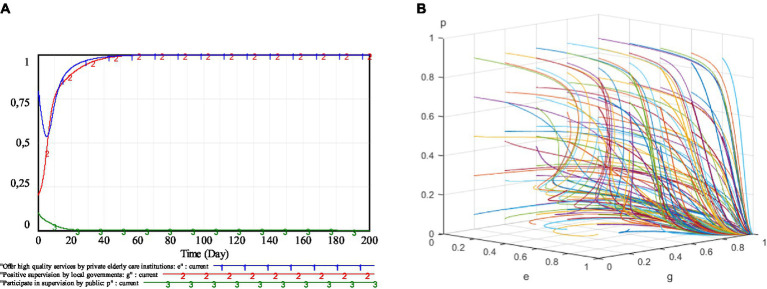
Simulation results under dynamic penalty and dynamic subsidy mechanism. **(A)** (*e, g, p*) = (0.79, 0.2, 0.1); **(B)** Different initial willingness.

Let the initial willingness of the three game subjects be (0.79, 0.2, 0.1), and set FINALTIME=200. The simulation results are shown in Figure 8. From Figure 8, it can be seen that, under the dynamic penalty and dynamic subsidy mechanism, the strategy choices of private elderly care institutions, local governments and the public will stabilize at (1, 1, 0). The evolutionary trajectory of private elderly care institutions and local governments is relatively smooth, with no long-term recurring fluctuations. The private elderly care institutions reach a stable state on day 58 and there is no possibility of supplying low-quality services.

#### 4.3.1. Proposition 3

The dynamic penalty and dynamic subsidy mechanism can bring the strategy choice of each subject in the regulation of the elderly service market to a steady state and stabilize at the ideal stability point (1, 1, 0).

##### 4.3.1.1. Proof

Replacing *F_e_* and S in the three-dimensional replicator dynamic system (I) by P(e) and S(e) respectively, the three-dimensional replicator dynamic system (IV) is obtained as follows:

(13) $$\left\{ \begin{array}{l} F\left(e\right)=e\left(1-e\right)\left[R_{h}+R_{e}+gS\left(e\right)-R_{l}+L_{e}+gP\left(e\right)+\left(1-g\right)pP\left(e\right)\right]\\ F\left(g\right)=g\left(1-g\right)\left[R_{g}-C_{g}-eS\left(e\right)+L_{g}+\left(1-e\right)\left(1-p\right)P\left(e\right)\right]\\ F\left(p\right)=p\left(1-p\right)\left[\left(1-e\right)R_{p}-C_{p}\right] \end{array}\right.$$

It follows from the three-dimensional replicator dynamic system (IV) that, eight new equilibrium points *E*_1_*_c_*(0, 0, 0), *E*_2_*_c_*(0, 0, 1), *E*_3_*_c_*(0, 1, 0), *E*_4_*_c_*(1, 0, 0), *E*_5_*_c_*(0, 1, 1), *E*_6_*_c_*(1, 0, 1), *E*_7_*_c_*(1, 1, 0), and *E*_8_*_c_*(1, 1, 1) can be calculated. Substituting the equilibrium point *E*_7_*_c_* into the Jacobian matrix $J^{\prime \prime \prime },$ the equation (14) can be obtained. According to equation (14), the eigenvalues corresponding to equilibrium point *E*_7_*_c_* can be calculated as $\lambda_{1}=-0.07,$ $\lambda_{2}=-0.1,$ and $\lambda_{3}=-0.2$ respectively. Because of $\lambda_{1},\lambda_{2},\lambda_{3}<0,$ so equilibrium point *E*_7_*_c_* is ESS. Similarly, equilibrium points *E*_1_*_c_* ~ *E*_6_*_c_* and *E*_8_*_c_* are not ESS.

(14) $$J^{\prime \prime \prime }=\left[\begin{array}{ccc} -0\mathrm{.07} & 0 & 0\\ 0 & -0\mathrm{.1} & 0\\ 0 & 0 & -0\mathrm{.2} \end{array}\right]$$

Proposition 3 suggests that, the dynamic penalty and dynamic subsidy mechanism not only restrains the fluctuation of the strategy selection of the three game subjects, but also speeds up the strategy evolution of the three game subjects and makes the system reach a more ideal stable state.

**Figure 9:**
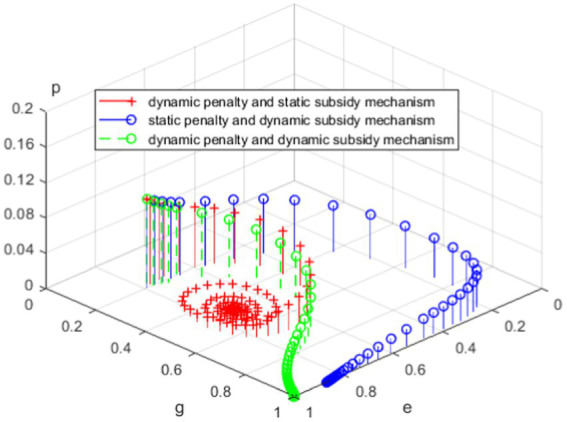
Evolutionary stability of three proposed mechanisms.

The above results suggest that the three regulatory mechanisms proposed can effectively compensate for the shortcomings of the static penalty and static subsidy mechanism. Comparing these regulatory mechanisms in Table 6 and Figure 9, we can find that the system cannot reach a stable state under the static supervision mechanism, while the system can stabilize at a certain point under the dynamic supervision mechanism. Furthermore, whether from the perspective of accelerating the evolution of the system to a stable state or from the perspective of improving the willingness of self-discipline management of private elderly care institutions, the dynamic penalty and dynamic subsidy mechanism is better than the static penalty and dynamic subsidy mechanism, and the static penalty and static subsidy mechanism is better than the dynamic penalty and static subsidy mechanism. That is to say, local government’s governance of the elderly services market is best under the dynamic penalty and dynamic subsidy mechanism. Therefore, on the one hand, local governments need to adopt dynamic penalty policies to effectively restrain speculation in the elderly service market; On the other hand, they need to adopt dynamic subsidy policies to reduce the operating costs of private elderly care institutions and fundamentally solve the problem of low-quality of the elderly services.

**Table 6:** Comparison of four supervision mechanisms.

Mechanism	Stable state	Time to reach steady state	Evolutionary stable strategy
Static penalty and static subsidy	Unstable	_	_
Dynamic penalty and static subsidy	Stable	180	(0.7201, 0.4725, 0)
Static penalty and dynamic subsidy	Stable	58	(0.869, 1, 0)
Dynamic penalty and dynamic subsidy	Stable	50	(1, 1, 0)

## 5. Sensitivity analyses

**Figure 10:**
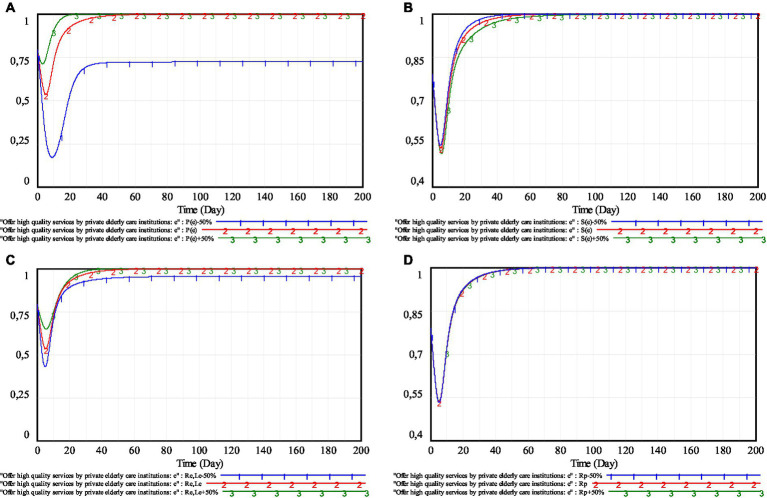
Simulation of the main parameters. **(A)** *P*(*e*); **(B)** *S*(*e*); **(C)** *R_e_*, *L_e_* and **(D)** *R*_*p*._

In order to discuss the impact of changes in the main parameters on the strategic choice of private elderly care institutions, the sensitivity analysis based on dynamic penalty and dynamic subsidy mechanism will be conducted. Furthermore, the priority of government policy adjustment in each scenario will also be analyzed. The simulation of the main parameters is shown in Figure 10.

As can be seen in Figure 10, as penalties and reputational gains-losses increase, private elderly care institutions will more actively choose to provide high-quality services. When the subsidies increase, the enthusiasm of private elderly care institutions to supply high-quality services will wane. This is because of that, the increased penalties and reputational gains-losses can increase the cost of speculation for private elderly care institutions and deter them from operating speculatively. The increase of subsidies will make the elderly service market which is not fully self-discipline get more subsidies. In this case, private elderly care institutions will collude. Some of them will choose to speculate, and others will continue to maintain self-discipline, in order to maximize the total subsidies received by the pension service market. The local governments’ rewards for the public supervision basically has little impact on the strategy choice of private elderly care institutions. That is, under the current public supervision willingness, the role of public supervision is limited.

For further study, the main parameters are compared in Table 7.

1. It can be seen from Table 7 that, under different parameter values, the minimum willingness of private elderly care institutions to provide high-quality services is lower than the initial willingness. Compared with subsidies and reputation gains-losses, the increase of penalties makes the minimum value of strategy choice of private elderly care institutions significantly increase. This implies that, increasing penalties can significantly improve the stability of the strategy evolution of private elderly care institutions. Therefore, if local governments are committed to improving market robustness, adjusting the penalties should be first considered.
2. It follows from Table 7 that, the speed of private elderly care institutions to reach a stable state is the fastest when the penalties and reputation gains-losses take a larger value, reaching a stable state on the 21st and 38th day, respectively. Moreover, the changes of penalties have the most obvious impact on the speed of private elderly care institutions to reach a stable state. The corresponding time difference is 61 days. Therefore, if the local governments are committed to speeding up market stability, adjusting the penalties should be first considered.
3. According to the difference between the final strategy choice ratio and the initial strategy choice ratio of private elderly care institutions in Table 7, both subsidies and supervision rewards can cause a large change in the strategy choice of private elderly care institutions when they take different values. When the supervision rewards apply different values, the evolutionary trajectory of private elderly care institutions does not change, and thus will not be considered. When the penalties and reputation gains-losses are small, the change of strategy choice of private elderly care institutions is also small, which are 0.0713 and 0.1631, respectively. Therefore, if local governments are committed to making the policy achieve better results, adjusting subsidies should be first considered.

**Table 7:** Comparison of main parameters.

Parameter	Value	Minimum value (time)	Time to reach steady state	Difference in time to reach steady state	Range of strategy changes
P(e)	P(e)−50%	e=0.1719(9)	82	$61^{***}$	$0.0713^{*}$
	P(e)	$e=0.5313{\left(5\right)}^{*}$	$57^{*}$		$0.21^{***}$
	P(e)+50%	$e=0.7188{\left(3\right)}^{***}$	$21^{***}$		$0.21^{***}$
S(e)	S(e)−50%	$e=0.5391{\left(5\right)}^{*}$	$43^{**}$	$45^{**}$	$0.21^{***}$
	S(e)	$e=0.5313{\left(5\right)}^{*}$	$57^{*}$		$0.21^{***}$
	S(e)+50%	$e=0.5156{\left(6\right)}^{*}$	88		$0.21^{***}$
*R_e_*, *L_e_*	$R_{e},L_{e}-50\%$	e=0.4297(5)	69	$31^{*}$	$0.1631^{**}$
	$R_{e},L_{e}$	$e=0.5313{\left(5\right)}^{*}$	$57^{*}$		$0.21^{***}$
	$R_{e},L_{e}+50\%$	$e=0.6563{\left(5\right)}^{**}$	$38^{***}$		$0.21^{***}$
*R_p_*	$R_{p}-50\%$	$e=0.5313{\left(5\right)}^{*}$	$57^{*}$	0	$0.21^{***}$
	$R_{p}$	$e=0.5313{\left(5\right)}^{*}$	$57^{*}$		$0.21^{***}$
	$R_{p}+50\%$	$e=0.5313{\left(5\right)}^{*}$	$57^{*}$		$0.21^{***}$

^*^, ^**^, and ^***^ be used to denote the priority of parameter regulation. ① For the minimum value, ^*^: 0.5 ≤ *e* ≤ 0.6, ^**^: 0.6 < *e* < 0.7, ^***^: *e* ≥ 0.7, other intervals are not marked. ② For the time to reach steady state, ^*^: 50 ≤ *d* ≤ 60, ^**^: 40 < *d* < 50, ^***^: *d* ≤ 40, other intervals are not marked. ③ For the difference in time to reach steady state, ^*^: 30 ≤ *d* ≤ 40, ^**^: 40 < *d* < 60, ^***^: *d* ≥ 60, other intervals are not marked. ④ For the range of strategy changes, ^*^: Δe≤0.1, ^**^: 0.1<Δe<0.2, ^***^: Δe≥0.2.

## 6. Discussion

In order to optimize the government’s supervision mechanism and improve the stability of the elderly service regulatory system, three supervision mechanisms, i.e., dynamic penalty and static subsidy, static penalty and dynamic subsidy, and dynamic penalty and dynamic subsidy, are proposed based on the tripartite evolutionary game model of private elderly care institutions, local governments, public. Using system dynamics theory, the evolutionary trajectories and steady states of the corresponding evolutionary equilibrium points under various mechanisms are then analyzed. The main suggestions are proposed as follows:

Firstly, the local governments’ policies on penalties and subsidies should be dynamically adjusted according to the behavior of private elderly care institutions. Under the static penalty and static subsidy mechanism adopted by the government, the service supply behavior of private elderly care institutions is uncertain. This mechanism will not only increase the financial burden of local governments, but also is not conducive to the healthy development of the elderly service market. Dynamic penalty and static subsidy mechanism, and static penalty and dynamic subsidy mechanism can make the strategy choice of private elderly care institutions reach a stable state, but private elderly care institutions still have a certain probability to choose to provide low-quality services. Only when the government adopts the dynamic penalty and dynamic subsidy mechanism, the behavioral evolution of private elderly care institutions can reach the most ideal state. Therefore, the government should pay close attention to the behavior of private elderly care institutions and make dynamic adjustments to the punishment and subsidy policies according to the behavior of private elderly care institutions.

Secondly, appropriately increase penalties, reduce subsidies, encourage media participation in reporting, and publicize assessment results. When the local governments adopt the dynamic penalty and dynamic subsidy mechanism, the self-discipline behavior of private elderly care institutions is positively correlated with the penalties and reputation gains-losses, and negatively correlated with the subsidies. Specifically, increasing penalties can restrain the speculation of private elderly care institutions, but excessive penalties are not conducive to market participants entering the elderly service industry. Increasing subsidies may make private elderly care institutions conspire to defraud the government of subsidies, which is not conducive to the improvement of the quality of elderly services. Therefore, penalties should be appropriately increased, subsidies should be reduced, and the level of reputational gains-losses should be increased through media engagement and publicity.

Finally, select appropriate parameters to adjust according to the government’s objectives. The government’s regulation of the elderly service market is often based on certain objectives, such as improving market robustness, speeding up market stability, or making the policy achieve better results. If the government’s regulatory goal is to improve market robustness or speed up market stability, the government should first adjust the punitive measures. If the government’s regulatory goal is to make the policy achieve better results, it will be an appropriate choice for the government to adjust the subsidies first.

The aforementioned statements are significantly different from the conclusions obtained by Ren et al. (51). In their paper, increased penalties are more conducive to the evolution of the system to the desired steady state when the government takes strict supervision as a long-term strategy, but they fail to consider that overly harsh regulation may not be conducive to the entry of market players. In addition, they neglect the influence of collusive behavior of service providers on subsidy strategy formulation, and thus they argue that subsidies help the system achieve the desired state under the cyclical subsidy mechanism. Also, the punishment and subsidy mechanism they proposed is static, which cannot be dynamically adjusted according to the behavior choice of service providers.

## 7. Conclusion

Using evolutionary game theory and system dynamics theory, the supervision of local governments on private elderly care institutions is discussed in this paper. The results show that: First of all, when the local governments adopt the static supervision mechanism, the game behaviors of private elderly care institutions, local governments, and the public cannot reach a stable state, while when the local governments adopt the dynamic supervision mechanism, the strategy choices of the three parties’ game subjects will reach a stable state. Secondly, among these dynamic supervision mechanisms, although the dynamic penalty and static subsidy mechanism, as well as the static penalty and dynamic subsidy mechanism can make the strategy choice of private elderly care institutions reach a stable state, private elderly care institutions still have a certain probability to choose to provide low-quality services. Only when the local governments adopt the dynamic penalty and dynamic subsidy mechanism, will the private elderly care institutions choose to operate with complete self-discipline. Moreover, under the dynamic penalty and dynamic subsidy mechanism, the willingness of self-discipline management of private elderly care institutions is positively related to penalties and reputation gains-losses, negatively related to subsidies, and not related to supervision rewards. Reasonable adjustment of these parameters can further optimize the dynamic penalty and dynamic subsidy mechanism. Thirdly, the government can first adjust penalties so that private elderly care institutions have greater stability in their strategic choices and reach stability more quickly. The government can also adjust the subsidies first to make the stability strategy of private elderly care institutions the most ideal.

Although the results of this paper are reasonable and meaningful, there are still some deficiencies in parameters design. In fact, taxation and consumer guidance are also important regulatory tools of government. For future research, therefore, the punishment, subsidy, taxation, and consumption guidance will be comprehensively considered, and the impact of the cross effects of various regulatory tools on the supervision of elderly care services will be analyzed.

## Data availability statement

The original contributions presented in the study are included in the article/supplementary material, further inquiries can be directed to the corresponding author.

## Author contributions

YZ and QW: conceptualization and methodology. QW: software, validation, formal analysis, writing—original draft, and visualization. YZ and JL: resources, writing—review and editing, and supervision. YZ: funding acquisition. All authors contributed to the article and approved the submitted version.

## Funding

This research was funded by the Anhui Provincial Education Department of China (Grant Number: SK2021ZD0049) and The Optimization Theory, Algorithms, and Applications (Grant Number: 2023SK104).

## Conflict of interest

The authors declare that the research was conducted in the absence of any commercial or financial relationships that could be construed as a potential conflict of interest.

## Publisher’s note

All claims expressed in this article are solely those of the authors and do not necessarily represent those of their affiliated organizations, or those of the publisher, the editors and the reviewers. Any product that may be evaluated in this article, or claim that may be made by its manufacturer, is not guaranteed or endorsed by the publisher.
